# The time course of altered brain activity during 7-day simulated microgravity

**DOI:** 10.3389/fnbeh.2015.00124

**Published:** 2015-05-12

**Authors:** Yang Liao, Meiying Lei, Haibo Huang, Chuang Wang, Jiaobo Duan, Hongzheng Li, Xufeng Liu

**Affiliations:** ^1^Department of Medical Psychology, Fourth Military Medical UniversityXi’an, Shaanxi, China; ^2^Mental Health Center, 303 Hospital of PLANanning, Guangxi, China; ^3^Department of Radiology, 303 Hospital of PLANanning, Guangxi, China

**Keywords:** head down tilt bed rest, simulated microgravity, self-adaption, brain activity, amplitude of low frequency fluctuation

## Abstract

Microgravity causes multiple changes in physical and mental levels in humans, which can induce performance deficiency among astronauts. Studying the variations in brain activity that occur during microgravity would help astronauts to deal with these changes. In the current study, resting-state functional magnetic resonance imaging (rs-fMRI) was used to observe the variations in brain activity during a 7-day head down tilt (HDT) bed rest, which is a common and reliable model for simulated microgravity. The amplitudes of low frequency fluctuation (ALFF) of twenty subjects were recorded pre-head down tilt (pre-HDT), during a bed rest period (HDT0), and then each day in the HDT period (HDT1–HDT7). One-way analysis of variance (ANOVA) of the ALFF values over these 8 days was used to test the variation across time period (*p* < 0.05, corrected). Compared to HDT0, subjects presented lower ALFF values in the posterior cingulate cortex (PCC) and higher ALFF values in the anterior cingulate cortex (ACC) during the HDT period, which may partially account for the lack of cognitive flexibility and alterations in autonomic nervous system seen among astronauts in microgravity. Additionally, the observed improvement in function in CPL during the HDT period may play a compensatory role to the functional decline in the paracentral lobule to sustain normal levels of fine motor control for astronauts in a microgravity environment. Above all, those floating brain activities during 7 days of simulated microgravity may indicate that the brain self-adapts to help astronauts adjust to the multiple negative stressors encountered in a microgravity environment.

## Introduction

Long before the first spaceship was sent into space, scientists had shown great interest in how the human body would change under that extreme environment. At the present time, more than five hundred astronauts around the world have accomplished hundreds of instances of spaceflight, allowing researchers to gain boundary knowledge on the issue mentioned above. According to previous studies, astronauts do not just enjoy the fantastic experience of space flight; they face simultaneous physical and psychological challenges induced by microgravity (De La Torre et al., 2012). These physical and psychological changes were assumed to be related to performance deficiency, which is a core problem in need of solutions (Eddy et al., 1998; Manzey et al., 2000; Mallis and DeRoshia, 2005; Zhao et al., 2011; Jiang et al., 2013; Koppelmans et al., 2013; Wang et al., 2013). Clarifying these physical and psychological changes during microgravity would help researchers to develop countermeasures to sustain an astronaut’s normal performance level, which would be of great importance to successful spaceflight missions.

In order to figure out the mechanism for the physical and psychological changes induced by microgravity, many studies attempted to clarify the negative effects of microgravity on human organs such as muscles, bones, and blood circulation (Grigoriev and Egorov, 1992; Pavy-Le Traon et al., 2007; Nicolas and Weiss, 2009; Williams et al., 2009; Moore et al., 2010). In contrast, the brain, as the control center of the human body, has received limited attention. In fact, the brain plays a key role in controlling behavior and cognition, which in turn affect performance. In this case, clarifying the brain activity changes induced by microgravity would greatly enrich our knowledge of the negative effects of microgravity. However, only a few previous studies have reported brain activity changes under real microgravity. Cheron et al. once observed increased power of the spontaneous 10 Hz oscillation in the parieto-occipital (alpha rhythm) and sensorimotor areas (mu rhythm) when astronauts were in spaceflight (Cheron et al., 2006). Cheron et al. proposed that this alpha power increase may be attributed to a change in brain blood oxygenation in real microgravity, and they assumed that these changes may in turn disturb the representation of space.

Obtaining high quality brain activity data in-orbit is difficult; brain activity data u real microgravity has rarely been reported. Instead, researchers use alternative methods, such as parabolic flight and head down tilt bed rest (HDT), to create a simulated microgravity environment, which also provides credible experimental evidence. Parabolic flight can create a 0 g condition for 20–30 s one time, and space agencies around the world prefer to use this technique to carry out quantitative research. Vladimir Pletser firstly reported variations of electroencephalographic (EEG) signals during different g levels in a parabolic flight study. These observations suggested that the altered EEG signals reflect the change of subject fatigue, which may affect the subject’s performance (Pletser and Quadens, 2003). Later on, Schneider et al. observed an inhibition of beta-2 EEG signals in the frontal area when subjects were exposed to simulated microgravity during parabolic flight. The researchers suggested that these brain activity changes were evoked by emotional fluctuations that may affect central nervous and adaptation processes (Schneider et al., 2008). Unlike in-orbit studies, these parabolic flight studies tend to ignore or deny the role of blood oxygenation variation in brain activity change. This difference may be due to the different lengths of the microgravity period between the two methods. From this viewpoint, HDT bed rest, which could create a continuous simulated microgravity environment, would provide similar and reliable evidence to the in-orbit environment. Dieter Vaitl et al. first reported inhibition of brain activity after 23 h HDT bed rest; they attributed this brain activity change to a body fluid shift (Vaitl et al., 1996). Later, Simone Messerotti Benvenuti et al. observed changed delta band EEG signals after 3 h HDT bed rest; the authors supposed this cortical inhibition was regulated by the baroreceptors’ deconditioning function (Messerotti Benvenuti et al., 2011). These EEG studies shed light on the relationship between brain activity change and blood oxygenation variation under the microgravity environment. But due to the low space resolution and low time resolution of the EEG method, the assumed relationship between brain activity changes and blood oxygenation variation is still not accurate enough.

Advanced imaging methods such as resting-state functional magnetic resonance imaging (fMRI) directly measure spontaneous neuronal activity (SNA) by recording the blood oxygen level-dependent signal, allowing researchers to gain more accurate data to find out the precise effect of microgravity on brain activity. Yang Liao et al. tentatively used resting-state fMRI to investigate brain activity changes after a 3-day HDT bed rest, the researchers observed decreased local activity in the thalamus and decreased regional homogeneity in the left inferior parietal lobule, which may be attributed to reduced motor control and decreased mental transformation abilities (Liao et al., 2012, 2013). Recently, Yuan Zhou, Shan-Guang Chen and Shu Li et al. carried out ground-breaking research to discuss the effects of long-term microgravity on the functional architecture of the brain. In this 45-day HDT bed rest study, a functional network anchored in the anterior insula and middle cingulated cortex was found to be influenced by simulated microgravity. The researchers suggested that these functional anomalies may reflect variations in cognitive function, autonomic neural function and central neural activity (Zhou et al., 2014). These imaging studies furthered our insight into the decreased performance of astronauts in microgravity, and they similarly described the cumulative effects of microgravity in different time spans. But the time trend of the effects of microgravity is still unknown; a sensitive and reliable brain function indicator is needed. Zang et al. proposed an improved approach to detect the amplitude of low-frequency fluctuation (ALFF), which is a reliable indicator of the extent of SNA (Zang et al., 2007; Zou et al., 2008; Cui et al., 2014). In our previous study, ALFF was verified as a sensitive indicator of brain activity changes in simulated microgravity, which meets the technical requirements of the current study (Liao et al., 2012).

In the current study, we carried out a 7-day 6° HDT bed rest experiment to simulate the microgravity environment. The resting-state fMRI data of all the participants were recorded before HDT bed rest and in every bed rest experiment day. ALFF was used to detect local brain activity changes, which are a sensitive and reliable indicator of the effects of microgravity on human brain over time. According to the above design, the current study may provide more details on the time pattern of brain activity changes induced by microgravity and facilitate our understanding of performance decline in astronauts.

## Materials and Methods

### Participants

Twenty male participants were recruited for this study. The valid participants’ mean age was 24 years, with a range from 20 to 32 years. All participants were right-handed, as measured by the Handedness Questionnaire. The participants reported no history of neurological injury, genetic mental disorders or substance abuse. With high-resolution T1- and T2-weighted MRI examination, no participant was observed to have significant pathological changes in their brain. The study was approved by the Ethical Committee of the Fourth Military Medical University and all participants provided their written informed consent before the experiment.

### HDT Bed Rest Procedure

The experiment was separated into three periods: that prior to bed rest (HDT0), the bed rest period (HDT1-HDT7) and the post bed rest period (7 days after bed rest, HDT14). Resting-state fMRI scans were taken on 19:00 every experimental day, which means every participant agreed to undergo imaging scans 9 times in total. During the HDT bed rest, adequate water and food were supplied, but the participants’ heads were prevented from moving from the bed to keep the redistribution of the individual’s body fluids toward the head. The experimental room was air-conditioned, and the temperature was maintained around 22°C. All of the testers are well educated with medical knowledge and skills, thus they could provide nursing care to the participants. Additionally, the participants were paired, and they were allowed to communicate with each other and engage in recreational activities (such as reading, watching television or movies, and using the internet) in their leisure time.

### MRI Data Acquisitions

The imaging data were collected by an experienced radiologist with an Achieva 3.0 T scanner in the radiology department of the 303rd Hospital of the People’s Liberation Army. The participants were instructed to lie flat on the scanning platform and place their heads in the assigned location. Sponge earplugs were used to plug the participant’s ears to minimize noise interference, and foam padding was used to restrict their head motion. A standard 8-channel head coil was used to collect data. The participants were instructed to keep their heads motionless, to remain calm with their eyes closed, and to not think of anything in particular. The scanning sequence and parameters used the following settings: the anatomical images of each location were T1-weighted images, TR = 3.1 ms, TE = 1.4 ms, matrix = 112 × 112; a three-dimensional turbo field echo (TFE) sequence was used for the high-definition three-dimensional T1-weighted images of the whole brain, TR = 7.6 ms, TE = 3.5 ms, flip angle (FA) = 8°, FOV = 250 mm × 250 mm, matrix = 512 × 512, slice thickness = 0.6 mm with no gap, and 301 slices; and a gradient echo planar imaging sequence was used to acquire functional images, TR = 2000 ms, TE = 35 ms, FA = 90°, FOV = 230 mm × 230 mm, matrix = 96 × 93, slice thickness = 4 mm, and 36 slices with no gap. This section of the scan contained 200 time points and lasted for 7 min.

### Data Preprocessing

The Data Processing Assistant for Resting-State fMRI (DPARSF)^1^ V2.0 was used for data preprocessing (Chao-Gan and Yu-Feng, 2010). The preprocessing steps were as follows. Initially, the data format of the functional images was transformed from DICOM to NIFTI. Second, because the participants required time to adjust, the initial ten time points were discarded, and 190 time points remained. Third, the left functional images were slice-time corrected and aligned with the initial image of each session for motion correction. No participants were excluded from the analyses because of excessive head motion (more than 2 mm or 2 degrees on any axis). Fourth, the data were then spatially normalized with the Montreal Neurological Institute (MNI) template (resampling voxel size = 3 × 3 × 3 mm^3^) in the Resting-State fMRI Data Analysis Toolkit (REST)^2^ V1.0 (Song et al., 2011). Fifth, the linear trend was removed, and the fMRI data were temporally band-pass filtered (0.01 ≤ f ≤ 0.08 Hz) to reduce the low-frequency drift and physiological high-frequency respiratory and cardiac noise. The resulting data were then spatially smoothed (4 mm FWHM Gaussian kernel).

### Analysis and Statistic for ALFF

The REST was used to perform the ALFF analysis. The procedure is similar to that used in previous studies (Yang et al., 2007). The filtered time series was transformed to a frequency domain using a fast Fourier transform (FFT) (parameters: taper per cent = 0, FFT length = shortest), and the power spectrum was obtained. Because the power of a given frequency is proportional to the square of the amplitude of the frequency component, its square root was calculated at each frequency of the power spectrum, and then the averaged square root was obtained across 0.01–0.08 Hz for each voxel. This averaged square root was considered the ALFF. For standardization purposes, the ALFF of each voxel was divided by the global mean ALFF value within a default brain mask in REST. To explore the ALFF differences among different HDT days, a one-way analysis of variance (ANOVA) was performed on the normalized ALFF maps with REST. The resultant statistical map was set at a combined threshold of *p* < 0.005 with a minimum cluster size of 351 mm^3^ (13 voxels), which corresponded with the corrected threshold of *p* < 0.05 determined by AlphaSim.^3^ The resulting map of the one-way ANOVA was saved as a Mask for *post hoc* *t*-tests by MRIcron (Rorden et al., 2007). Then *post hoc* *t*-tests were performed to identify the difference between each day of the HDT period and the pre-HDT day by using the above-mentioned MASK (*p* < 0.05 determined by AlphaSim). Then the for presentation purposes, the statistical maps were superimposed on the higher-resolution anatomical template available in REST and the MNI coordinates of peak *t*-value were transformed to Talarich coordinates with the REST Slice Viewer (a utility tool in REST).

## Results

**Figure 1:**
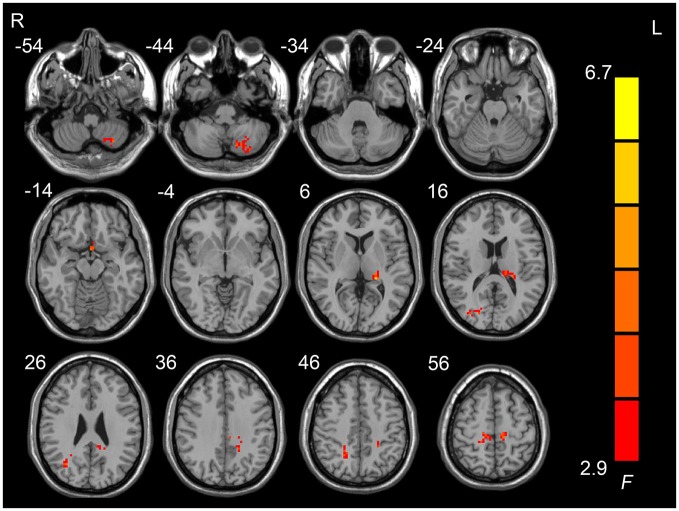
**Regions showing amplitudes of low frequency fluctuation (ALFF) differences across the pre-HDT period and HDT period**.

Figure 1 shows the one-way ANOVA analysis of the ALFF values in the pre-HDT period and each day of the HDT period. Regions showing significant ALFF differences between these 8 days were the left cerebellum posterior lobe, the left paracentral lobule, the left anterior cingulate, the left superior frontal gyrus, the left limbic lobe, the left thalamus, the right lingual gyrus, the right postcentral gyrus and the right middle temporal gyrus (*p* < 0.05, corrected).

**Figure 2:**
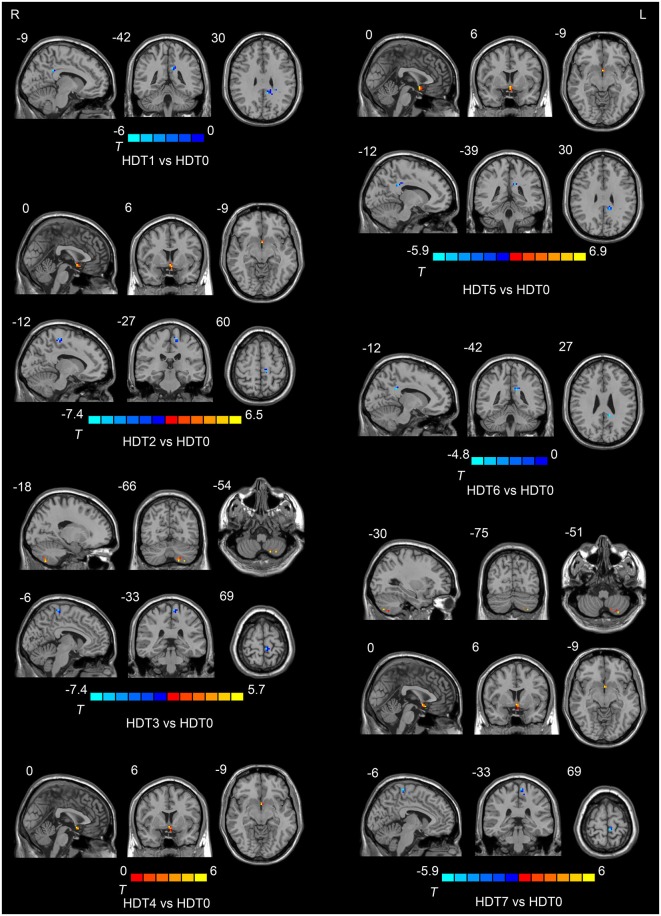
**Regions showing ALFF differences among the pre-HDT period and each days in HDT period**.

Compared to HDT0, subjects presented lower ALFF values in the posterior cingulate cortex (PCC) on HDT1 and HDT5 (*p* < 0.05, corrected) and lower ALFF values in the left paracentral lobule on HDT2, HDT3 and HDT7 (*p* < 0.05, corrected). Relative to HDT0, subjects exhibited higher ALFF values in the anterior cingulate cortex (ACC) on HDT2, HDT4, HDT5, and HDT7 (*p* < 0.05, corrected), and they presented higher ALFF values in the left cerebellum posterior lobe (CPL) on HDT3 and HDT7 (*p* < 0.05, corrected) (Table 1; Figure 2).

**Table 1:** **Regions showing ALFF differences among the pre-HDT period and each days in HDT period**.

Region	Brodmann area	Voxels	MNI coordinate			Peak-*t* value
			*X*	*Y*	*Z*
HDT1 vs. HDT0
Posterior cingulate cortex	31	21	−9	−42	30	−6.06
HDT2 vs. HDT0
Anterior cingulate cortex	25	18	0	6	−9	6.46
Left paracentral lobule	6	21	−12	−27	60	−5.03
HDT3 vs. HDT0
Left cerebellum posterior lobe		23	−18	−66	−54	5.41
Left paracentral lobule	6	20	−6	−33	69	−7.49
HDT4 vs. HDT0
Anterior cingulate cortex	25	14	0	6	−9	6.01
HDT5 vs. HDT0
Anterior cingulate cortex	25	21	0	6	−9	6.85
Posterior cingulate cortex	31	14	−12	−39	30	−5.98
HDT6 vs. HDT0
Posterior cingulate cortex	31	15	−12	−42	27	−4.80
HDT7 vs. HDT0
Left cerebellum posterior lobe		20	−30	−75	−51	5.41
Anterior cingulate cortex	25	16	0	6	−9	6.02
Left paracentral lobule	6	26	−6	−33	69	−5.98

## Discussion

Previous studies have demonstrated that ALFF can reflect the extent of SNA; therefore, the altered ALFF observed in our study could deepen the understanding of brain activity changes induced by microgravity (Bing et al., 2013; Liu et al., 2014; Yu et al., 2014). In the current study, changes in ALFF were found in the PCC, the left paracentral lobule, the ACC and the left cerebellum posterior lobe. These brain areas play vital roles in adjusting cognitive functions and corresponding performance.

In the current study, decreased activity in PCC was found during HDT bed rest. As previous studies indicated, PCC plays a critical role in both the default mode network and the dorsal attention network (Brewer et al., 2013; Molnar-Szakacs and Uddin, 2013). Though the relationships between PCC and these brain networks are still unclear, abundant published studies have reached a consensus that PCC plays an important role in attention (Weissman et al., 2006; Li et al., 2007; Margulies et al., 2009; Leech et al., 2011; Hinds et al., 2013; Wen et al., 2013). One hypothesis is that PCC is involved in noticing internal and external changes and facilitating novel behavior or thought in response. Low activity in this region would indicate continued operation with the current cognitive set and is not beneficial for the flexible adjustment of cognition and behavior in changing situations (Pearson et al., 2011). An alternative hypothesis is that PCC plays a crucial role in controlling the state of arousal, the breadth of focus, and the internal or external focus of attention. The decreased activity in the PCC may in turn disrupt the normal functioning of attention (Leech and Sharp, 2014). Despite the differences between the two hypotheses, one thing we know is that suppressed activity in the PCC could damage attention, either in attention flexibility or attention span. In this regard, the decreased activity in the PCC after HDT may partially account for the lack of cognitive flexibility observed in astronauts in microgravity.

Additionally, activity in the paracentral lobule during HDT bed rest was also found to be lower than the pre-HDT bed rest period. The paracentral lobule could be divided into two sub regions, the anterior portion of the paracentral lobule is part of the frontal lobe and is often referred to as the supplementary motor area while the posterior portion is considered part of the parietal lobe and deals with somatosensation of the distal limbs. The decreased paracentral lobule activity observed in the current study was mainly located in the anterior portion, which is involved in the control of movement (Ritter et al., 2009). A number of studies focused on human motor function in microgravity reported diminished fine motor control among astronauts, and the decreased activity in the paracentral lobule found in the current study may partly explain this phenomenon. In contrast, increased activity in the CPL was observed during HDT bed rest. Based on anatomical knowledge, the CPL mainly receives input from the brainstem and cerebral cortex; recent studies have shown that this region plays an important role in fine motor coordination (Siegel and Sapru, 2006; Stoodley and Schmahmann, 2010; Stoodley, 2012; Stoodley et al., 2012). From the view of neurobiology, the CPL could inhibit involuntary movement via inhibitory neurotransmitters (especially GABA) (Voogd and Glickstein, 1998). Interestingly, the increased activity in the CPL seemed to have the same time trend as the decreased activity in the paracentral lobule during HDT bed rest, which may indicate that the improvement function in the CPL plays a compensatory role to the functional decline of the paracentral lobule in a microgravity environment.

Additionally, increased activity in the ACC was found during the HDT bed rest period. The ACC is a sub-region of the ventromedial frontal cortex and plays multiple complex roles in brain. Among healthy subjects, increased ACC activation has been linked with a series of cognitive and emotional functions, such as attention, conflict or performance monitoring, autonomic nervous system control, and homeostatic incongruence. The ACC is active when challenging physical conditions disturb homeostasis (Tataranni et al., 1999; Freedman et al., 2006; Bie-Olsen et al., 2009; von Leupoldt et al., 2009). In a changing environment, homeostatic incongruence would be disturbed and the demands of attention and executive control would increase to full fill the need to respond to complex and novel stimuli. The increased activity in the ACC found in a simulated microgravity environment may indicate a reaction in the central nervous system corresponding to alterations in autonomic nervous system activity, which was designed to counteract the increased demands of cognition. This finding is consistent with previous studies (Zhou et al., 2014).

Two alternative hypotheses could explain the brain activity changes observed in the current study. The first hypothesis focuses on the direct physical effects of body fluid changes induced by the absence of gravity. Previous studies have proved that astronauts’ body fluids would transfer cephalad in microgravity, as a result of the self-adjustment of body fluid circulation to gravity change. Some researchers speculated that this physiological effect may change the blood flow in the brain and thus affect the brain activities in some way. An alternative hypothesis is that the brain activity change is a psychological stress process resulting from varying gravity. While astronauts were initially exposed to a microgravity environment, their performance would sharply descend due to stressors such as the absence of gravity. After several days of adaptation, their performance would restore to normal levels. Though these two hypotheses are very different from each other, the first hypotheses underlines the physiological effects of body fluid change while the latter emphasizes the psychological effects of stress, they could both partly explain the brain activity changes observed in the current study. Based on the data observed in the current study, we could not clarify which hypothesis is right. In fact, physical change and psychological change could interaction with each other, thus these two hypotheses are not exclusive.

Overall, the floating brain activities during 7-day simulated microgravity may indicate that brain self-adaption was motivated to help astronauts to adapt to the absence of gravity in a microgravity environment. Those findings in the simulated microgravity environment may improve our understanding of the time trend of physical and psychological changes in the variation of gravity, but we should still be cautious about extended these results to the real microgravity environment.

## Author and Contributions

Authors XL, HL and ML conceived and designed the experiments. Authors YL, HH and CW performed the experiments. YL analyzed the data. JD contributed materials and analysis tools. Authors YL and HH wrote the manuscript and contributed equally to this work.

## Conflict of Interest Statement

The authors declare that the research was conducted in the absence of any commercial or financial relationships that could be construed as a potential conflict of interest.

## Footnotes

^1^http://www.restfmri.net/forum/DPARSF

^2^www.restfmri.net

^3^http://afni.nimh.nih.gov/pub/dist/doc/manual/AlphaSim.pdf
